# Disrespect during childbirth and postpartum mental health: a French cohort study

**DOI:** 10.1186/s12884-023-05551-3

**Published:** 2023-04-12

**Authors:** Emma Leavy, Marion Cortet, Cyril Huissoud, Thomas Desplanches, Jessica Sormani, Sylvie Viaux-Savelon, Corinne Dupont, Swann Pichon, Laurent Gaucher

**Affiliations:** 1 Geneva School of Health Sciences, HES-SO University of Applied Sciences and Arts, Western Switzerland47, av. de Champel, Geneva, CH-1206 Switzerland; 2 Hospices Civils de Lyon, Hôpital de la Croix-Rousse, Lyon, F-69004 France; 3 Hospices Civils de Lyon, Hôpital Femme Mère-Enfant, Bron, F-69500 France; 4 Réseau Périnatal AURORE, Lyon, F-69004 France; 5 Research on Healthcare Performance (RESHAPE), Université Claude Bernard Lyon 1, INSERM U1290, Lyon, F-69008 France

**Keywords:** Mental Health, Post-Traumatic Stress Disorders, Depression, Postpartum, Attitude of Health Personnel, Delivery, Obstetric

## Abstract

**Background:**

While the World Health Organisation (WHO) warned about mistreatment, disrespect and/or abuse during childbirth as early as 2014. This same year a social media movement with #payetonuterus brought to light the problematic of obstetrical violence in French speaking countries, and more specifically on issues of disrespect. The experience of care is an integral part of the quality of care, and perception on inadequate support during labour and loss of control in labour are some of the most frequently reported risk factors for childbirth-related post-traumatic stress disorder (CB-PTSD). Therefore, it seems crucial to study the associations between disrespect during childbirth and the mental well-being of mothers.

**Methods:**

We performed a multicentered cohort study using auto-questionnaires within a French perinatal network. The main outcome was women’s report of disrespect during childbirth measured by the *Behavior of the Mother’s Caregivers – Satisfaction Questionnaire* (BMC-SQ) 3 days and 2 months after childbirth. CB-PTSD and Postpartum Depression (PPD) were assessed 2 months after childbirth using respectively the *Post-Traumatic Checklist Scale* (PCLS) and the *Edinburgh Postnatal Depression Scale* (EPDS).

**Results:**

This study followed 123 mothers from childbirth to 2 months postpartum. Among them, 8.13% (n = 10/123) reported disrespect during childbirth at 3 days after childbirth. With retrospect, 10.56% (n = 13/123) reported disrespect during childbirth at 2 months postpartum, i.e. an increase of 31%. Some 10.56% (n = 13/123) of mothers suffered from postpartum depression, and 4.06% (n = 5/123) were considered to have CB-PTSD at 2 months after childbirth. Reported disrespect during childbirth 3 days after birth was significantly associated with higher CB-PTSD 2 months after birth (R^2^ = 0.11, F(1,117) = 15.14, p < 0.001 and β = 9.11, p = 0.006), PPD at 2 months after childbirth was positively associated to reported disrespect in the birth room, 3 days after birth (R^2^ = 0.04, F(1, 117) = 6.28, p = 0.01 and β = 3.36, p = 0.096). Meanwhile, PPD and CB-PTSD were significantly associated 2 months after childbirth (R^2^ = 0.41, F=(1,117) = 82.39, p < 0.01 and β = 11.41, p < 0.001).

**Conclusions:**

Disrespect during childbirth was associated with poorer mental health during the postpartum period. Given the high prevalence of mental health problems and the increased susceptibility to depression during the postpartum period, these correlational results highlight the importance of gaining a deeper awareness of healthcare professionals about behaviours or attitudes which might be experienced as disrespectful during childbirth.

**Supplementary Information:**

The online version contains supplementary material available at 10.1186/s12884-023-05551-3.

## Background

In 2014, the World Health Organisation (WHO) alerts on the need to prevent mistreatment, disrespect and abuse during childbirth, especially in low-income countries where facility-based childbirth are increasing [1]. It is during the same year the slogan #payetonuterus (i.e. literally “get your uterus”, a call to denounce mistreatment, disrespect and/or abuse during gynaecological care) emerged on Twitter social media in France to point out bad experiences of childbirth and perinatal care. A year later, the International Federation of Gynecology and Obstetrics defined 7 categories of disrespect and abuse during childbirth also known as obstetric violence (i.e. physical abuse, non-consented care, non-confidential care, non-dignified care, discrimination based on specific patient attributes, abandonment of care, and detention in facilities) and agreed to take actions to reduce them [2].

While countries with advanced healthcare systems may not feel concerned by the problem, a raise in awareness emerged first on social media in 2014 with the French hashtag #payetonuterus. This was a popular movement among French speaking women which highlighted the inappropriate gestures or words used by healthcare professionals and especially gynaecologists. A more global wave of hashtags became particularly noticeable in 2017, the #MeToo. This was an encouraging movement for women to speak up about all inappropriateness going from inappropriate comments to sexual assaults. There was an international wave of testimonies of women reporting having suffered from obstetric violence [3]. This has been raising awareness in last few years among healthcare workers and in research regarding the importance of respectful care, especially at a vulnerable time such as during childbirth. Considering that physical violence and mistreatment were an epiphenomenon in France, the French National Academy of Medicine (*Académie Nationale de Médecine*) has focused only on the disrespects committed by the caregivers and has not retained the overall constituents of obstetric violence. The WHO’s definition regarding obstetric violence and mistreatment covers these dimensions, but also includes other elements which aren’t contextually relevant to the French context, such as “sterilization, detention of women and their new-borns, refusal of admission to health facilities, …”. The French National Academy of Medicine distinguishes 7 dimensions of disrespect during childbirth (i.e. attitude, respect for privacy, language, and/or gentleness of care and procedures, information provided, women’s participation in decision-making, and/or consideration of pain) [4, 5].

According to the WHO, high quality care during childbirth includes “optimum skills and attitude of health providers” [6]. Perception on inadequate support during labour and loss of control in labour are some of the most frequently reported risk factors for CB-PTSD after childbirth [7]. Hence, the experience of care during childbirth and in particular the perception of (dis)respectful behaviours and attitudes of healthcare workers are important aspects of care which may have potential long-term negative consequences on mental health.

In this framework, we intend to carry out a French cohort pilot study to document and understand the association between mother’s experience of disrespect during childbirth measured at 3 days postpartum and her postpartum mental health assessed through the levels of post-traumatic stress and depression following childbirth, both measured at 2 months postpartum.

## Methods

### Recruitment, and participants

From September 2018, we conducted a multicentre observational cohort study measuring the women’s report of disrespect during childbirth and evaluating their mental health during postpartum. The study was conducted in postpartum wards of the 25 French maternity units covered by the AURORE perinatal network (i.e., 5% of all births in France). Women were included in the study if aged 18 years old or more, spoke French and had given birth to viable and a live-born child. Women were excluded if they had lost the child in the few days after birth or refused to participate at the 2 months survey. On a total of 627 respondents, a sample of 123 women agreed to participate up to 2 months postpartum and completed all the required measures.

Only 15 of the 25 maternity units in our network had birthing rooms intended for physiological births, 12 of them equipped with a bathtub. Even when they are available, they are rarely used due to lack of human resources, with midwives usually attending two or three women in labour simultaneously. In France in 2021, 69% of maternity hospitals reported that they had a space dedicated to physiological births, but that the number of midwives was insufficient to allow for one-to-one support [8]. Only 21% of the maternity hospitals authorized the presence of independent midwives to assist women with a physiological birth project. Consequently, most births that occur in these units have high levels of medical management, including induction of labour (26%) and epidural pain relief (83%). Births ended with a caesarean in 21% of cases, with instrumental extraction in 12% and with non-instrumental vaginal births in 66%. Midwives attend 89% of non-instrumental vaginal births [8].

### Study design and outcome measures

The primary outcome was the report of disrespect during childbirth using the Behavior of the Mother’s Caregivers – Satisfaction Questionnaires (BMC-SQ). This self-administered questionnaire distinguishes 7 dimensions of disrespect during childbirth (i.e. attitude, respect for privacy, language, and/or gentleness of care and procedures, information provided, women’s participation in decision-making, and/or consideration of pain). As the French National Academy of Medicine suggests, if one of these dimensions is reported as disrespectful by the mother, then it is considered that the mother is victim of disrespect [4, 5]. It was adopted and validated in French language by the AURORE network (Cronbach’s alpha 0.92) [5]. This measure was taken at 3 days postpartum.

Our secondary outcomes were the level of CB-PTSD and PPD, both measures were taken at 2 months postpartum. The level of post-traumatic stress following childbirth was assessed using the Post-Traumatic Checklist Scale (PCLS). The PCLS is a self-administered questionnaire of 17 items including multiple-choice questions answered on a Likert-type scale containing four responses scored from 1 to 5 [9]. Women were considered to have CB-PTSD at 2 months if their PCLS scores exceeded 44 (expected sensitivity of 97% and specificity of 87%) as recommended by previous authors [10]. The level of PPD was assessed using the Edinburgh Postnatal Depression Scale (EPDS), a 10-item self-administered scale with multiple-choice questions on a Likert-type scale containing four responses scored from 0 to 3 [11]. Women were considered to have PPD if their EPDS scores met the threshold of a score greater than or equal to 11. At two months, the EPDS cut-off score and percentage of women who were considered to be clinically depressed was calculated, with an expected sensitivity of 80% and an expected specificity of 92% as recommended by previous research [11].

Finally, research midwives collected characteristics of participants from their medical records, including their social, demographic, and clinical characteristics (i.e., maternal age, pre-pregnancy body mass index, region of birth, educational level, parity, multiple pregnancy, history of hospitalization for depression), childbirth factors (i.e., woman transferred before childbirth, term of birth, mode of admission to birth room, mode of birth, perineal tears or lacerations, analgesia, infant birthweight, breast feeding initiation, neonatal resuscitation, and transfer to the neonatal intensive care unit), and characteristics about the maternity units at which each birth took place (i.e., university or not, public or private, and annual volume of births).

### Statistical analysis

All statistical analyses were performed with the R software, version 4.2.1 [12]. Quantitative variables with normal distributions according to the Shapiro–Wilk test were described by their means and standard deviations (SD), and then compared with a Welch two-sample t-test. When distributions were not normal, variables were described according to their medians, with their 25th and 75th percentiles (Q1–Q3), and then compared by a Wilcoxon rank sum test. Qualitative variables were described as the number of individuals and percentages and then compared with Fisher’s exact test. The denominator is reported when it comprises less than 95% of the total sample size.

Two multiple linear regression allowed us to observe the associations between reported disrespect during childbirth initially reported (i.e. during the first 3 days following childbirth), EPDS scores, PCLS scores, and other characteristics. These mental health related scores were entered as dependent variables in R and all other characteristics as independent variables. We report the results of a regression analysis according to the American Psychological Association (APA) recommendations, including the coefficient of determination (R^2^), the F value, the degrees of freedom in parentheses, the β, and the p-value.

## Results

**Fig. 1:**
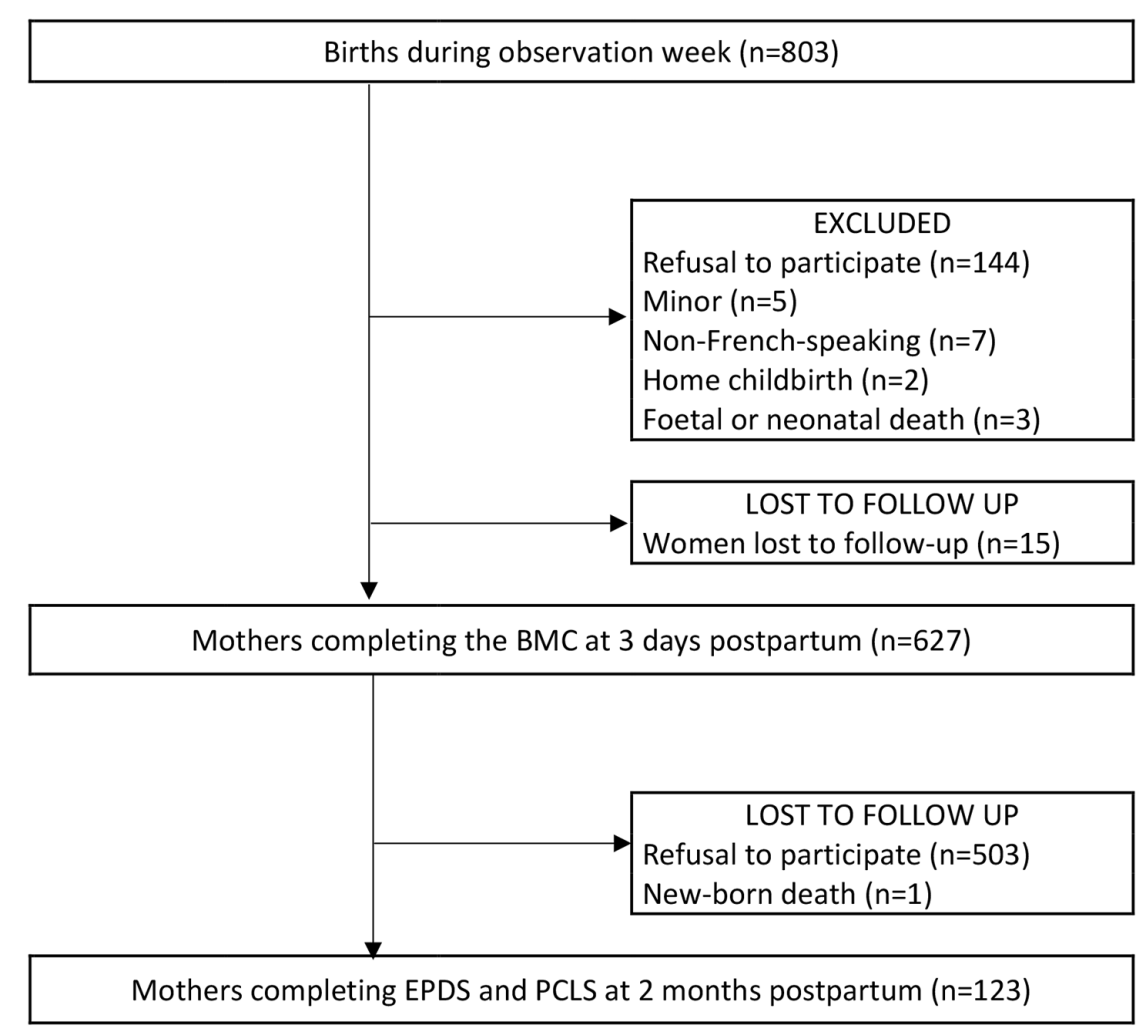
Study flow chart

During the week of the study, 123 mothers agreed to participate (Fig. 1). These respondents had a mean age of 31 years (SD = 3.9), and 45.5% were nulliparous (n = 56/123). Most gave birth at term (n = 117/123, 95.1%), vaginally (n = 103/123, 83.7%), with regional analgesia (n = 100/123, 81.3%), in public maternity units (n = 82/123, 66.7%) (Table 1).

The evaluation of our primary outcome showed that 8.1% (n = 10/123) of women reported disrespect during childbirth within the first 3 days after birth, and a higher proportion did so (10.6%, n = 13/123) when questioned at 2 months post-partum (Table 1). The dimension in which mothers reported the highest incidence of disrespect during childbirth was the lack of consideration for pain, with 5.69% (n = 7/123) at 3 days after birth and 9.76% (12/123) at 2 months (Supplementary material, Table S1). Reporting of inappropriate attitudes or behaviour by caregivers increased from 2.4% (n = 3/123) when reported in the maternity wards following birth, and up to 6.5% (n = 8/123) when at home 2 months postpartum, representing an increase of 267% (Table S1).

Regarding our secondary outcomes, 10.9% (n = 13/123) of respondents were considered depressed (i.e. EPDS score ≥ 11) at 2 months postpartum, and 4.2% (n = 5/123) considered to have CB-PTSD at 2 months postpartum (i.e. PCLS score > 44). Reported disrespect during childbirth in the 3 days following childbirth was positively associated with higher levels of CB-PTSD score at 2 months postpartum (R^2^ = 0.11, F(1,117) = 15.14, p < 0.0001 and β = 9.11, p = 0.006). Notably, 1.8% (n = 2/109) of the women not reporting disrespect at D3 had a pathological PTSD score at 2 months compared to 30.0% (n = 3/10) in the group of women reporting disrespect (p = 0.004). PPD at 2 months was also positively associated with the report of disrespect during childbirth 3 days after childbirth, although this association was marginally significant (R^2^ = 0.04, F(1,117) = 6.28, p = 0.01 and β = 3.36, p = 0.096) (Table 2). Finally, PPD at 2 months was positively associated with CB-PTSD at 2 months (R^2^ = 0.41, F(1,117) = 82.39, p < 0.01 and β = 11.41, p = 0.001).

**Table 1:** Characteristics of mothers who participated in study up to 2 months postpartum

Characteristics	Participants (n = 123)
Age in years, mean (SD)	31.4 (3.9)
Body mass index, median [IQR]	24.1 [5.4]
Weight gain during pregnancy, mean (SD)	11.2 (6.0)
Country of birth, n (%)	
France	118 (95.9)
Other European	1 (0.8)
Other non-European	4 (3.3)
Postsecondary education ≥ 2, n (%) (%)years, n (%)	97 (79.5)
History of depression, n (%)	1 (0.8)
Primiparous, n (%)	56 (45.5)
Preterm Birth, n (%)	6 (4.9)
Beginning of labour, n (%)	
Spontaneous	93 (75.6)
Prophylactic caesarean	10 (8.1)
Labour induction	20 (16.3)
Mode of birth, n (%)	
Non-instrumental	90 (73.2)
Instrumental	13 (10.6)
Prophylactic caesarean	10 (8.1)
Emergency caesarean	10 (8.1)
Perineal trauma, n (%)	
None	54 (43.9)
1st degree tears	45 (36.6)
2nd degree tears	23 (18.7)
3rd degree tears	1 (0.8)
4th degree tears	0 (0.0)
Episiotomy	8 (6.5)
Epidural analgesia, n (%)	100 (81.3)
New-born hospitalized, n (%)	3 (2.4)
Breastfeeding	99 (82.1)
Healthcare facility	
Private not-for-profit	19 (15.4)
Public university	20 (16.3)
Public non-university	62 (50.4)
Private	22 (17.9)
Annual number of deliveries, n (%) (%)	
< 1000	16 (13.0)
1000–2000	55 (44.7)
2000–3000	29 (23.6)
> 3000	23 (18.7)
Victim of disrespect during childbirth	
Reported 3 days postpartum	10 (8.1)
Reported 2 months postpartum	13 (10.6)
PCLS score, median [25–75 perc.]	20.0 [17.0–25.0])
EPDS score, median [25–75 perc.]	4.0 [2.0–8.0]

**Table 2:** Multiple linear regressions of independent factors predicting either total PCLS or EPDS Scores measured 2 months after birth (n = 123)

		PCLS Scores R^2^ = 0.39, F(25,75) = 1.91, p < 0.05			EPDS Scores R^2^ = 0.23, F(25,75) = 0.88, p > 0.1		
**Predictors**		*Estimated β*	*CI*	*P*	*Estimated β*	*CI*	*P*
Age in years	[18–25]	Reference (0.00)			Reference (0.00)		
	[26–35]	-1.05	-8.57 to 6.46	0.780	-1.86	-6.50 to 2.78	0.427
	[36–45]	0.32	-8.52 to 9.17	0.942	-1.39	-6.86 to 4.07	0.613
BMI before pregnancy	< or equal to 25	Reference (0.00)			Reference (0.00)		
	> 25	2.01	-2.27 to 6.29	0.353	1.25	-1.39 to 3.89	0.351
Weight gain during pregnancy	< or equal to 16 kg	Reference (0.00)			Reference (0.00)		
	> 16 kg	3.42	-2.61 to 9.46	0.262	2.04	-1.68 to 5.77	0.278
Country of birth	France	Reference (0.00)			Reference (0.00)		
	Other European countries	-6.21	-25.55 to 13.12	0.524	2.48	-9.45 to 14.42	0.679
	Non-European countries	1.01	-9.59 to 11.61	0.849	4.21	-2.34 to 10.75	0.205
Postsecondary education	< 2 years	Reference (0.00)			Reference (0.00)		
	> 2 years	-1.09	-6.09 to 3.91	0.665	0.26	-2.82 to 3.36	0.862
History of hospitalisation for depression	No	Reference (0.00)			Reference (0.00)		
	Yes	10.37	-7.84 to 28.58	0.260	4.46	-6.78 to 15.70	0.43
Birth term	> 37 WG	Reference (0.00)			Reference (0.00)		
	< or equal to 37 WG	7.11	-3.78 to 18.02	0.197	0.96	-5.77 to 7.69	0.777
Mode of delivery	Non-instrumental	Reference (0.00)			Reference (0.00)		
	Instrumental	0.17	-7.25 to 7.59	0.964	1.09	-3.78 to 5.09	0.634
	Emergency caesarean	-2.23	-9.41 to 4.95	0.538	0.65	-3.47 to 5.67	0.769
Beginning of labour	Spontaneous	Reference (0.00)			Reference (0.00)		
	Labour induction	-4.84	-11.04 to 1.36	0.124	-0.31	-4.13 to 3.52	0.874
Perineal tear	None	Reference (0.00)			Reference (0.00)		
	Degree 1	-5.34	-9.81 to -0.88	0.019	-1.37	-4.13 to 1.38	0.324
	Degree 2	-7.38	-12.51 to -2.26	0.005	-2.27	-5.43 to 0.89	0.157
Episiotomy	No	Reference (0.00)			Reference (0.00)		
	Yes	-8.52	-15.80 to -1.24	0.022	-2.22	-6.72 to 2.27	0.327
Analgesia	Yes	Reference (0.00)			Reference (0.00)		
	No	-2.74	-7.37 to 1.89	0.243	0.18	-2.68 to 3.05	0.898
Outcome for newborns	Stay with mother	Reference (0.00)			Reference (0.00)		
	Hospitalization	6.39	-6.11 to 18.89	0.312	1.80	-5.91 to 9.52	0.643
Breast feeding	Exclusive	Reference (0.00)			Reference (0.00)		
	Artificial	-2.58	-7.92 to 2.76	0.339	-1.32	-4.61 to 1.98	0.428
	Mixed	-2.86	-15.28 to 9.57	0.648	-2.44	-10.11 to 5.22	0.527
Healthcare facility	Private not-for-profit	Reference (0.00)			Reference (0.00)		
	Public university	-4.91	-3.16 to 11.46	0.545	-2.32	-12.25 to 7.61	0.643
	Public non-university	4.79	-1.66 to 11.25	0.143	1.76	-2.22 to 5.74	0.382
	Private hospital	4.15	-3.16 to 11.46	0.261	2.94	-1.57 to 7.46	0.197
Number of annual deliveries	< 1000	Reference (0.00)			Reference (0.00)		
	1000–2000	-3.09	-9.67 to 3.47	0.351	-1.02	-5.08 to 3.04	0.617
	2000–3000	-2.63	-9.08 to 3.81	0.418	-1.32	-5.29 to 2.66	0.511
	> 3000	6.59	-8.67 to 21.85	0.392	3.01	-6.41 to 12.44	0.526
Victim of disrespect during childbirth	No	Reference (0.00)			Reference (0.00)		
	Yes	9.36	2.86 to 15.86	0.005	3.48	-0.53 to 7.49	0.088

## Discussion

### Main findings

Early report of disrespect during childbirth by caregivers was positively associated with higher levels of CB-PTSD 2 months following childbirth, and – although not significantly – positively associated with PPD, 2 months after birth. With a two-month delay, the proportion of mothers reporting having experienced disrespect during childbirth was higher than reports right after birth. This difference seemed especially marked for reports targeting the disrespectful attitudes or behaviours of caregivers during birth. Women who had suffered from perineal tears or episiotomy had less severe post-traumatic stress scores, knowing that all the perineal tears were almost exclusively simple 1st or 2nd degree tears.

### Strengths and limitations

The originality and main strength of this study is the repeated measurement of disrespect during childbirth, which allowed us to investigate how the perception by mothers of disrespect evolves 2 months after childbirth. However, the French definition of disrespect is more restrictive than those of mistreatment, abuse or obstetrical violence. Unfortunately, as in any online postpartum survey, the participation rate at 2 months was low. However, the characteristics of the included mothers seem to be homogeneous with those of the French population, which suggests a limited selection bias [8]. A recent cross-sectional study had already identified an association between obstetric violence and the mental condition of mothers, but to our knowledge, our study is the first cohort to confirmed this association [13]. Although a probable lack of power prevents us from concluding on the association between disrespect during childbirth and depression, our power was sufficient to demonstrate a link with CB-PTSD.

### Interpretation

Our study shows significant association between reported disrespect during childbirth in the day following childbirth and CB-PTSD at 2 months postpartum. This finding is in line with earlier studies in high-, middle- and low-income countries. For example a study based in India, women reporting negative childbirth experience is associated to higher risks for CB-PTSD 6 weeks after childbirth [14] and such as another study based in Spain were obstetric violence is also associated to mental conditions [13]. Within different socioeconomic backgrounds such as India, women reporting negative childbirth experience show associated higher risks for CB-PTSD 6 weeks after childbirth [14]. This emphasizes the need for respectful care during childbirth, regardless of the socioeconomic background.

After 2 months, the proportion of reported disrespect during childbirth increased. One Danes and one Swedish study also reported increased dissatisfaction of mothers throughout time; women’s assessment shortly after birth compared to a considerable period after, is reported more negatively [15, 16]. More worryingly, a Japanese study found that the risk of loss to follow-up (i.e. non-response to questionnaires) in the postpartum period was associated with psychological distress in mothers [17]. This raises the question of finding the best way and the best time to ask mothers about their experience of birth.

Although our sample doesn’t let us bring any conclusions regarding non-French women, we may highlight the need to bring more attention to reported violence of non-French women considering that a recent study in Denmark suggests that mental health needs are less assessed among women with migratory background, due to cultural and linguistic differences [18].

Surprisingly, we observed the practice of episiotomy is significantly associated with lower levels of CB-PTSD, compared to women with no episiotomy. On the contrary, other studies find obstetric interventions such as episiotomy to be a risk factor of CB-PTSD [19–21]. These findings continue to worry health researchers and professionals which bring growing interest to CB-PTSD, PPD and their associated risk factors [21–24].

### Research recommendation

Several systematic reviews were conducted to identify risk factors associated with CB-PTSD, but there is a lack of data to understand how care practices might impact on the risk of developing CB-PTSD. In addition, the experience component of care practice, including the recent concept of disrespect, mistreatment, or abuse by caregivers (i.e. obstetric violence) has emerged as a factor that could potentially be associated with CB-PTSD as we found in this pilot study. But, future research should be conducted longitudinally to investigate these associations. The collection of outcomes reported by women via online questionnaires may be an obstacle to consider, as well as the different languages in which these questionnaires should be offered. One specific symptom of CB-PTSD is the avoidance of stimuli related to childbirth, the source of the trauma, which lead women to postpone their medical follow-up. This avoidance of care is an additional concern with potential major consequences on women’s and their families’ health, as well as on health costs. Data on the economic costs are lacking and call for further research.

### Practical recommendation

Given the positive link we observed between women’s reports of obstetric disrespect during childbirth and subsequent CB-PTSD, a first implication for clinical practice would be to raise healthcare staff’s sensitivity to both issues of direspect during care and to the issue of CB-PTSD. These issues could be measured and health care workers trained. Another implication will be to highlight the importance of maintaining contact with mothers over a longer period, e.g., at least 6 months postpartum [25]. Also, healthcare workers must give further attention to women who declare dissatisfaction regarding the care they received during childbirth. Regarding the cost that such a recommendation may imply, the use of new technologies such as tele-health services during the postpartum period may help to reduce the subsequent burden of PPD and CB-PTSD. Tele-consultation has indeed already shown to be effective for reducing prenatal stress and anxiety [26]. On the one hand, such measure would help to better record listen and accompany victims of obstetrical disrespect. On the other hand, it would also help to detect and better handle the subsequent development of PPD or CB-PTSD in women, many of which often remain untreated. Finally, there are structural causes of obstetric violence that need to be addressed [27]. One perspective of improvement for France might be the development of midwives-led birth units, who currently cover less than 1% of births.

## Conclusions

We found that reported disrespect during childbirth was positively associated with CB-PTSD, which was significantly associated with PPD. These findings bring questions among healthcare workers regarding the importance of respectful and appropriate care during childbirth. On the other hand, the increased reports in obstetrical disrespect 2 months after birth highlights the need for future studies which investigate obstetrical disrespect to quantify these measures over a longer period of time, as well as their potential consequences on the development of CB-PTSD.

## Electronic supplementary material

Below is the link to the electronic supplementary material.

**Additional file 1: Table S1**. Number of women?s assessments (and % total) of disrespect during childbirth by caregivers

## Data Availability

The data that support the findings of this study are available from the corresponding author upon reasonable request.

## Abbreviations

BMC-SQ: Behavior of the Mother’s Caregivers – Satisfaction Questionnaire
PPD: Postpartum Depression
PTSD: Post-Traumatic Stress Disorder
EPDS: Edinburgh Postnatal Depression Scale
PCLS: Post-Traumatic Checklist Scale
