# Supplementary material for: Disrespect during childbirth and postpartum mental health: a French cohort study
Source: BMC Pregnancy Childbirth. 2023 Apr 12;23:241. doi: 10.1186/s12884-023-05551-3 (PMC10091597; doi:10.1186/s12884-023-05551-3)
Supplement: Supplementary file 1 — Additional file 1: Table S1. Number of women?s assessments (and % total) of disrespect during childbirth by caregivers [file 12884_2023_5551_MOESM1_ESM.docx]

Table S1. Number of women’s assessments (and % total) of disrespect during childbirth by caregivers

|  | **Participants at 3 days postpartum**  **(n=123)** | | | | **Participants at 2 months postpartum**  **(n=123)** | | | |
| --- | --- | --- | --- | --- | --- | --- | --- | --- |
|  | *Completely* | *Sufficiently* | *Insufficiently* | *Not at all* | *Completely* | *Sufficiently* | *Insufficiently* | *Not at all* |
| Appropriate attitude or behaviour | 105 (85.37) | 15 (12.19) | 3 (2.44) | 0 (0.00) | 91 (73.98) | 24 (19.51) | 7 (5.69) | 1 (0.81) |
| Respect for privacy | 95 (77.24) | 28 (22.76) | 0 (0.00) | 0 (0.00) | 91 (73.98) | 31 (25.20) | 1 (0.81) | 0 (0.00) |
| Appropriate language | 103 (83.74) | 19 (15.45) | 1 (0.81) | 0 (0.00) | 90 (73.17) | 28 (22.76) | 5 (4.06) | 0 (0.00) |
| Gentleness of care | 97 (78.86) | 26 (21.14) | 0 (0.00) | 0 (0.00) | 92 (74.79) | 29 (23.58) | 2 (1.62) | 0 (0.00) |
| Clear and appropriate information | 98 (79.68) | 23 (18.70) | 2 (1.62) | 0 (0.00) | 91 (73.98) | 30 (24.39) | 2 (1.62) | 0 (0.00) |
| Consideration of pain | 91 (73.98) | 25 (20.33) | 7 (5.69) | 0 (0.00) | 87 (70.73) | 24 (19.51) | 12 (9.76) | 0 (0.00) |
| Participation in decision-making | 94 (76.42) | 26 (21.15) | 2 (1.62) | 1 (0.81) | 87 (70.73) | 32 (26.01) | 3 (2.44) | 1 (0.81) |
